# Fine-Scale Dissection of Functional Protein Network Organization by Statistical Network Analysis

**DOI:** 10.1371/journal.pone.0006017

**Published:** 2009-06-24

**Authors:** Kakajan Komurov, Mehmet H. Gunes, Michael A. White

**Affiliations:** 1 Department of Cell Biology, University of Texas Southwestern Medical Center, Dallas, Texas, United States of America; 2 Department of Computer Science and Engineering, University of Nevada, Reno, Nevada, United States of America; Dalhousie University, Canada

## Abstract

Revealing organizational principles of biological networks is an important goal of systems biology. In this study, we sought to analyze the dynamic organizational principles within the protein interaction network by studying the characteristics of individual neighborhoods of proteins within the network based on their gene expression as well as protein-protein interaction patterns. By clustering proteins into distinct groups based on their neighborhood gene expression characteristics, we identify several significant trends in the dynamic organization of the protein interaction network. We show that proteins with distinct neighborhood gene expression characteristics are positioned in specific localities in the protein interaction network thereby playing specific roles in the dynamic network connectivity. Remarkably, our analysis reveals a neighborhood characteristic that corresponds to the most centrally located group of proteins within the network. Further, we show that the connectivity pattern displayed by this group is consistent with the notion of “rich club connectivity” in complex networks. Importantly, our findings are largely reproducible in networks constructed using independent and different datasets.

## Introduction

Dynamic architecture of the protein interaction network has an important role in the regulation of cell behavior. Understanding the functional organization of protein interaction networks is of utmost importance for our understanding of the principles regulating cellular behavior and consequently in understanding the diseases where cellular behavior is misregulated.

Accumulation of biological data through large-scale genomics and proteomics and the introduction of mathematical and computational tools have launched a quest for deciphering principles governing the organizational framework of protein networks. Several previously characterized notions from statistical physics and computer science regarding network topology have been adapted into systems biology in order to explain the functional organization of protein networks [1]–[5]. However most of these studies have considered the protein interaction networks without taking into account the dynamic nature of protein expression, which is essential for a proper representation of biological networks. In addition, some of these notions have been met with criticisms in the field [6]–[9], underlining the non-trivial nature of the organization of biological networks and the need for more rigorous analyses in gaining insight into the functional organization of protein networks.

In order to gain an in-depth understanding of the dynamic organization of the protein interaction network and its role in the regulation of cellular processes, we derived several graph theoretical metrics in order to capture the dynamic expression properties of proteins as well as of their neighborhoods (i.e. set of interacting partners in the network). Using these metrics, we identified several classes of proteins with distinct dynamic expression profiles (dynamical classes). We show that each of these dynamical classes has specific roles in the connectivity of the protein interaction network, regulation of cell behavior or both. Among these classes, we identify one with the most central positioning in the network and reveal a special connectivity pattern of proteins in this group that is important for the robust regulation of signaling within the cell. Importantly, our findings on the dynamic organization of the protein network are consistent across two other independent interaction datasets. Finally, we show that our analysis can resolve the discrepancy between recent reports regarding the dynamic modularity in the protein interaction network by providing a more in-depth view of the protein network organization.

## Results

In order to account for the dynamic properties of proteins as well as their dynamic relationship with their neighbors in the network, we used gene expression information from a large compendium of microarray data and a high quality collection of protein interaction data to derive 9 network metrics that describe the dynamic behavior of a protein and of its neighborhood in the network (see Methods). Briefly, we defined expression variance (EV) in order to capture the variability of a protein's expression across multiple conditions, neighborhood EV in order to describe the neighborhood of a protein in terms of their EV, neighborhood EV variance (*v^EV^*) to account for variability of EVs of neighbors of a protein, average interactor Pearson correlation coefficient (avPCC) to describe how a protein is co-regulated with its neighbors, neighborhood PCC (nPCC) to ask if the neighbors of a protein are co-expressed with each other, nPCC2 to describe co-expression of proteins in the second neighborhood of a protein, neighborhood PCC variance (*v^PCC^*) to account for variability of expression profiles of proteins in the neighborhood, dynamic degree (*yK*) to ask if a protein is co-regulated with other proteins in the network, and neighborhood *yK* (*nyK*) to account for average *yK* in the neighborhood. These metrics are explained in detail in the Methods. Collectively, these metrics define a dynamic profile for each protein.

### Classification of proteins according to their dynamic profiles in the network

**Figure 1:**
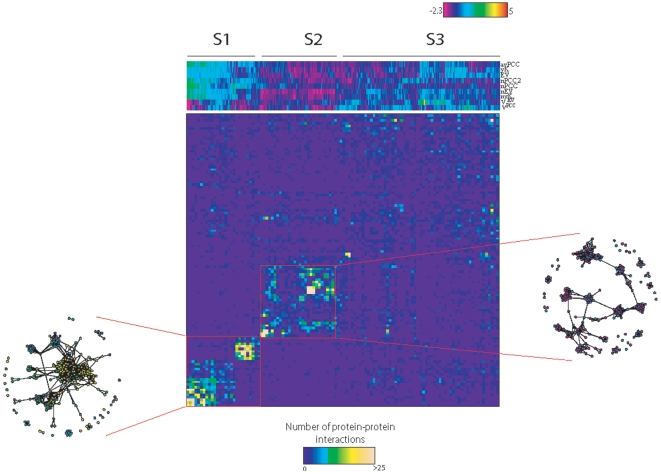
Dissection of proteins into dynamical classes. Hierarchical clustering of proteins, using Ward's method, by their dynamic profiles (upper panel) and the interaction matrix showing the protein-protein interaction patterns between different dynamic profiles (lower panel). In order to make clustering possible, we normalized each row to have a mean of 0 and a variance of 1. For the lower panel, proteins were binned into 114 bins with the exact ordering as in the clustering in the upper panel. Each square in the matrix represents the number of interactions between respective bins.

First, a dynamic profile (values based on each metric) was assigned to each protein in the network based on these metrics. Then, we performed a hierarchical clustering of proteins in order to identify distinct classes of dynamic profiles in the network and to test if they represent specific functions of proteins in the network. We only evaluated highly connected proteins (i.e. those that have >6 interaction partners, which is the upper 30^th^ percentile of the node degree distribution), as they produced best clustering with these values when compared to the clustering performed by proteins having lower node degrees (not shown). From the graphical representation of the clustering, it is possible to dissect three main groups of proteins (Fig. 1). Group S1 is characterized by the highest nPCC, avPCC, nEV and EV values, while S2 has the lowest values in these categories. An obvious distinguishing feature of S1 and S2 from the group S3 is their lower *v^EV^* values, indicating that S1 and S2 proteins are located in neighborhoods with homogeneous expression profiles. Despite having higher variation in terms of most values, S3 proteins consistently have higher *v^EV^* values, suggesting that these proteins are located within highly variable neighborhoods.

In order to get a first impression about the connectivity profiles of these groups in the network, we examined the protein-protein interactions of these groups within and between each other. For this purpose, we binned the proteins into 114 bins of 10 proteins, respecting the order of proteins in the clustering above, and calculated the number of interactions between every bin pair. Strikingly, we see a significant interaction density within the S1 and S2 groups, but not in S3 or between S1 and S2, immediately suggesting the existence of densely connected clusters in these groups (Fig. 1a). Indeed, a network plot of these groups reveals densely connected clusters of high and low EVs respectively (Fig. 1a), indicating that the groups S1 and S2 are mainly composed of highly co-regulated dynamic and non-variant static densely connected clusters of proteins.

Densely connected clusters are likely to represent specialized modules in the cell [10]. Indeed, S1 proteins have significantly higher nPCC and nPCC2 values, which indicate that S1 proteins represent dynamically expressed modules. In addition, these proteins have higher nEV and EV values, pointing to their highly dynamic expression pattern. S2 proteins, on the other hand, have the lowest EV, nEV, *v^EV^*, *v^PCC^*, nPCC and nPCC2 values, which strongly suggests that these proteins are located in neighborhoods with non-variant expression patterns. Static neighborhoods have been shown to be highly specialized functional modules [9]. Therefore, S1 and S2 groups represent dynamic and static modules, respectively.

Of S1, S2 and S3, dynamic profiles of S3 proteins are the most disparate. The only common characteristic of proteins in this group seems to be the almost invariant high *v^EV^* or *v^PCC^* values, which excludes these proteins from modules, where expression properties of proteins are similar. The disparity of the dynamic profiles of these proteins may stem from the versatility of their functions, as they are located more centrally in the network (as judged from their betweenness centrality scores, not shown) and therefore may have functions in multiple processes. However, a close analysis of the clusters generated by hierarchical clustering of S3 reveals subgroups of proteins with distinct dynamic profiles (Text S1). We hypothesized that these different dynamic profiles may correspond to different functional classes of S3 proteins and therefore analyzed them in more depth.

### Dynamic classes have distinct roles in network connectivity

In order to analyze if the dynamic profiles have distinct roles in network connectivity, we separated S1 and S3 groups into more subgroups based on their dynamic profiles (see Text S1). These classes are distinguished from each other by one or more characteristics that give insights about the dynamic nature of their neighborhood and suggest specific functions that these proteins may be performing in their respective localities in the network (see Text S1). For example, separation of S1 into 3 subgroups suggests that there are two subclasses of dynamic modules, those with high EV and those with lower EV (see Text S1). Interestingly, there seems to be a functional distinction even between these two, high EV dynamic modules being almost exclusively those involved in ribosomal RNA synthesis and processing as well as translation, whereas dynamic modules with lower EV are almost exclusively proteasomal complexes (not shown).

In order to analyze the specific roles of these dynamic classes in the organization of the protein network, we undertook an *in silico* loss-of-function approach where we remove the desired set of proteins from the network and observe where the connectivity has been perturbed in the network (see Methods). We removed each dynamic class of proteins from our network, and measured where the network path lengths of proteins has increased. An increase in the path length between two nodes *a* and *b* upon removal of a node *c* indicates that the node *c* lies on the path between nodes *a* and *b*. Here, we only measured changes in path lengths between proteins that are separated by one node in the original network (path length = 2). Thus, when we remove a group *c* of proteins from the network and see that the network paths from a group *a* of proteins to another group *b* of proteins has been increased, we conclude that the group *c* proteins are directly linking proteins of groups *a* and *b*.

**Figure 2:**
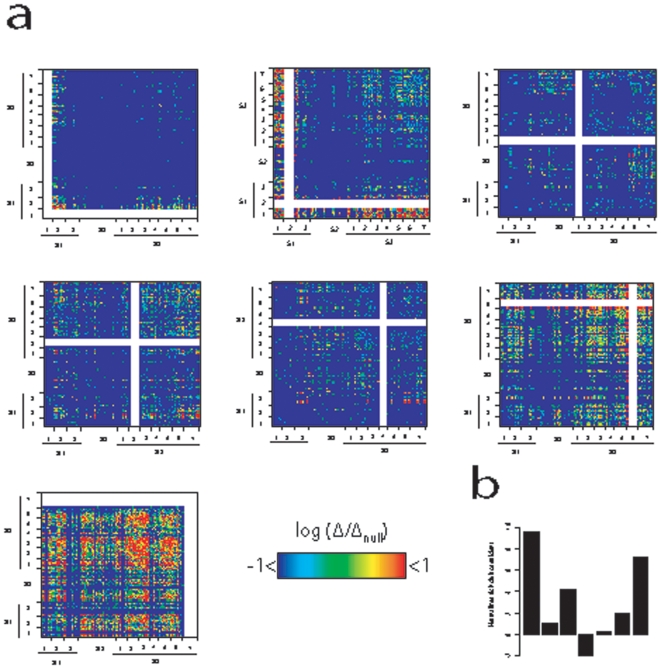
Characterization of roles of subgroups in the network connectivity. a) Deletion profiles of select subgroups. White stripes in the heatmaps indicate the deleted group. Please see Methods for a detailed description of the deletion profiles. b) Normalized rich club coefficients (see Methods) of each group.

Fig. 2a shows the results for the removal of each dynamical class from the network as compared to the removal of the same number of randomly selected proteins of similar node degrees. The removal of subgroup S1.1, which mainly contains ribosome biosynthesis dynamic modules, impairs the connection between S1.2 proteins as well as the connection of other proteins to S1.2. Removal of S1.2 has an even stronger impact on the connectivity of S1.1 proteins to each other as well as to most of the rest of the network. These results indicate that S1.2 and S1.1 proteins are inter-linked to each other and that S1.2 proteins are probably located between proteins S1.1 and most of the rest of the network, thereby bridging the two.

Removal of subgroups S1.3 and S2 does not seem to impact the connectivity of the network, corroborating with the idea that these proteins are isolated modules with highly specialized functions (not shown). Connections of S2 to S3.6 and S3.7 are however impaired by the removal of S3.1, which is in accordance with the dynamic profile of this subgroup, which shows that although these proteins are mainly surrounded by static proteins, they are also interacting with dynamic proteins (see Text S1). An important role of S3.1 proteins in the network may be in connecting the static modules to the rest of the network.

The dynamic profile of S3.2 suggests that these proteins interact with dynamic proteins that are not modular (see Text S1). Fig. 2a shows that their removal has the most significant impact on the connection of S1.2 to the proteins of S3.6 and S3.7, indicating that S3.2 proteins are coordinating the connections between proteins in S1.2 and proteins in S3.6 and S3.7.

The most significant feature of proteins in S3.4 is their high nPCC2 but low nPCC values, which suggest that these proteins are found “just outside” of dynamic modules (Text S1). Accordingly, their removal from the network results in an impaired connectivity between dynamic modules of S1.3 and the proteins in S3.6 and S3.7. Therefore, it can be concluded that S3.4 proteins are playing a role as coordinators of dynamic modules in S1.3.

Although the overall betweenness centrality values of groups S3.5 through S3.7 are not significantly different from each other (not shown), removal of each has markedly different effects on the network connectivity. While removal of S3.5 does not significantly affect the network connectivity when compared to randomly selected proteins (not shown), removal of S3.6 proteins seems to affect the connectivity of most of S3 proteins to each other as well as to S1.1 and S1.2 (Fig. 2a). However the most potent effect on the network connectivity is seen with the removal of S3.7, where connections within and between almost every group of proteins becomes impaired (Fig. 2a). This observation argues that S3.7 proteins may be the most centrally located proteins in the network as their deletion results in a severe network disorganization. Since our *in silico* loss-of-function approach only takes into account node pairs that are only 1 node apart in the original network (see above and Methods), the profile of S3.7 deletion may indicate that these proteins are highly dispersed throughout the network as opposed to more localized positioning of other groups. Given its significantly higher impact on the network connectivity as compared to other groups, we hypothesized that S3.7 may contain proteins that play roles as the central coordinators of cellular events, and therefore analyzed this group in more depth.

### S3.7: a “rich club” of central organizers in the network

Although deletion of S3.7 from the network results in a significantly greater disintegration of connectivity among other groups, S3.7 proteins are not significantly more centrally located in the network as judged from their betweenness, degree or closeness centralities (three metrics commonly used to measure a node's centrality in the network [11]) (not shown). This is surprising at first sight, because betweenness centrality of a node measures the frequency of paths between all node pairs that pass through that node, and Fig. 2a shows that the paths between most node pairs get impaired upon removal of S3.7 proteins. It is conceivable, therefore, that S3.7 proteins may not be as central individually as they are as a group. In order to test this, we calculated group betweenness values (measures the centrality of a group of proteins) of all groups, and find that S3.7 proteins have a more significant group betweenness than other S3 groups (not shown).

An observation that a group of nodes are significantly central as a group but not as individuals suggests that there is some redundancy among group members regarding connectivity of the network. This notion requires that the group members are tightly connected to each other so that the absence of one node would be compensated by another in the network. Indeed, a network plot of the dynamic classes shows that S3.7 has a considerable within-group interaction density as compared to others (Figure S1), which corroborates with a possibility of a within-group redundancy in terms of connectivity. Interestingly, among the S3 groups, only S3.1 and S3.7 seemed to be displaying significant overall within-group connectivity (Figure S1). In order to test if the observed density of interactions among S3.7 proteins is expected by chance, we compared within-group interaction densities of the groups with those in 100 randomized instances of the network, and find that S3.7 proteins are significantly more inter-connected than what would be expected by chance (Fig. 2b). Only S3.1 and S3.3 groups have within-group interaction densities close to that of S3.7. However unlike S3.7, where proteins are inter-linked to each other predominantly in a single connected web, S3.1 and S3.3 groups contain some proteins that form small dense clusters with each other, thus contributing to their high densities of within-group interactions. Therefore, it follows that S3.7 proteins form a well-connected web in the cellular network that regulates the connectivity among different classes of proteins. This specific connectivity pattern, where instead of being dispersed in the network, central proteins are tightly inter-connected in a web, resembles so-called “rich-club” connectivity pattern in social networks and may have important implications about the cellular mechanisms of regulating information flow within the protein network (see Discussion).

Another striking feature of S3.7 is that proteins in this group are highly regulated as evidenced from their high EV, but nevertheless are not subject to a significant co-regulation with other genes in the network as evidenced from their low *yK* (see Text S1), despite the high correlation between EV and *yK* (Spearman's ρ = 0.68). This indicates that S3.7 proteins are not likely to be a part of cellular gene expression programs and therefore have less constraint in their expression when compared to other high EV proteins (compare to S1, S3.5 and S3.6). This property may corroborate with the notion that these proteins are the central regulators of cellular processes (see Text S1). Accordingly, S3.7 contains the master regulators of processes like pheromone response: STE11, FUS3 and STE12; and cell cycle: SWI5, TEM1 and CKS1.

### Global dynamic layout of the protein interaction network

**Figure 3:**
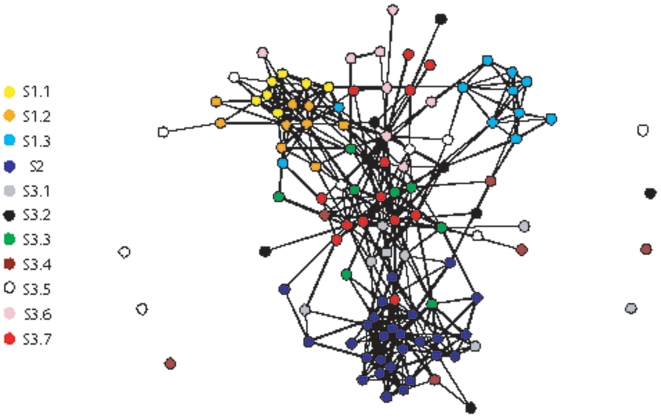
The reduced network plot. Each node in this network represents one of bins used to construct the interaction matrix in Figure 1. Therefore, each bin represents 10 proteins with most similar dynamic profiles. In this network, there is an interaction between two bins only if there are at least 4 number of protein-protein interactions between proteins in the two bins. Bins are colored according to which dynamic class they belong.

Next, in order to generate a visualization of the dynamic layout pattern across the network suggested by our analyses, we constructed a reduced network by using the bins used to construct the interaction matrix in Figure 1. Figure 3 shows the network of bins, where each interaction represents at least 4 protein-protein interactions between the proteins in respective bins (Figure 3). A clear organized pattern of the network is evident from this plot, where proteins with different dynamic profiles seem to be positioned within well-defined network localities relative to each other. It is possible to dissect three distinct classes of dense modules, static modules (S2), high EV dynamic modules (S1.1) and low EV dynamic modules (S1.3), with S1.2 connecting S1.1 to the rest of the network, as suggested by our *in silico* loss-of-function approach, and S3.4 coordinating the connections of S1.3 to most of other proteins in the network, which was also suggested by our *in silico* loss-of-function analysis. Importantly, the three classes of modules are each specialized for a specific process, high EV dynamic modules (S1.1) are those performing rRNA synthesis, processing and translation, low EV dynamic modules (S1.3) are those performing protesomal protein degradation, and static modules (S2) are those performing mRNA synthesis, splicing and transcriptional control. Other proteins in the network are positioned between these modules according to their dynamic profiles, possibly coordinating functions of these modules.

Most of S3.7 proteins are positioned in the center of this network and seem to be densely connected to each other, also in accordance with our observations above. This plot provides a graphically intuitive representation of our analyses presented above regarding the dynamic organization pattern within the protein interaction network.

### The dynamic organization pattern is reproducible across different datasets

**Figure 4:**
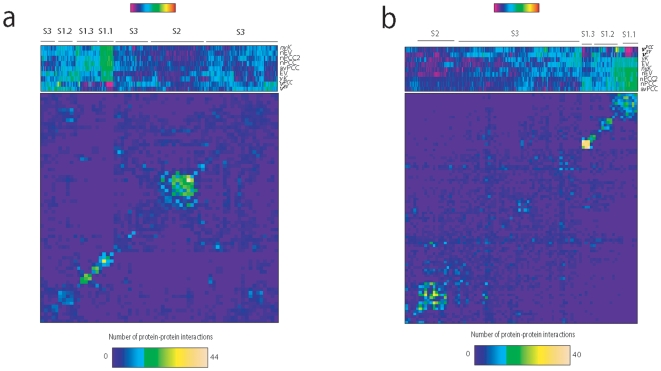
Heatmaps for the dynamic profiles of proteins in two independent datasets and their protein-protein interaction profiles. a) High confidence dataset from Bertins *et al* (2006). b) High confidence dataset from Batada *et al* (2006). Ordering of proteins in bins of the interaction matrices are exactly like in the heatmaps above each matrix. Clustering was done the same way as for our dataset (see text).

An important factor to be considered in protein network studies is the high rate of false positives in high throughput protein-protein interaction data. Even though our dataset contains only high quality data [12], [13], we wanted to check if the dynamic profiles in this study and their interaction profiles can be reproduced using other high quality datasets. For this purpose, we used high quality datasets from two recent studies that reported contradictory findings with respect to each other about network modularity [7], [14]. A clear separation of S1 and of its subgroups, S2 and S3 groups as well as their interaction patterns very similar to the one in Figure 1 can be seen in both datasets (Fig. 4). Out of each dataset, we extracted a cluster that most resembled S3.7 according to their dynamic profiles. Our criterion for S3.7 was that the cluster must have a high EV, low *yK*, low nPCC and avPCC, moderate nEV and high *v^EV^* or *v^PCC^*, in accordance with the profile of S3.7 (see Text S1). The resulting set of proteins had a significant overlap with S3.7 (p∼10^−5^, hypergeometric distribution), which suggests that this set is enriched for S3.7 proteins. In both datasets, the cluster we extracted had a significantly higher within-group density of interactions than what would be expected by chance (not shown), supporting our observations above.

Using their high quality dataset, Batada *et al* (2007) argued against the model of organized modularity in the protein interaction network [6], [7] that was proposed earlier [15]. Interestingly, using our approach, we show that their dataset in fact supports the model of organized dynamic modularity. We suggest that our multi-dimensional approach can resolve the discrepancy in literature by providing a more comprehensive view of the protein network characteristics.

## Discussion

In this work, we first derived several novel graph theoretical metrics to explain the dynamic behavior of a protein and of its neighborhoods in the network, and then out of a global distribution of dynamic profiles of proteins we revealed a highly specific and organized functional dynamic layout model of the protein network, which seems to be consistent across different datasets.

We had previously characterized static and dynamic modules [9]. In this work, by taking a more comprehensive approach, we confirm the existence of static and dynamic modules. In addition, we also identify a different subclass of dynamic modules that have lower EV values. Interestingly, this subgroup of dynamic modules is mainly composed of proteasomal complexes. It follows that high EV dynamic modules are mainly involved in protein synthesis (through rRNA synthesis and translation), while lower EV dynamic modules are specialized for protein degradation.

We have found that the most central organizers of the protein interaction network have a high preference of interactions for each other and therefore form a highly connected web at the core of the network. This connectivity pattern is reminiscent of the “rich club” phenomenon in complex networks, which is characterized by a significant connection density among “important” hubs (i.e. “rich” nodes) in the network (hence “rich club”), and has implications in the network routing efficiency, redundancy and/or robustness [16], [17]. The rich-club in the internet network has been suggested to serve as a super traffic hub and provide a large selection of shortcuts for a greater efficiency and flexibility of the traffic routing [16]. In the case of protein interaction networks, dense connectivity between central proteins may indicate fast communication between different parts of the network and/or a highly coordinated control of cell behavior through dense within-group interactions.

Another dimension to this intriguing scenario is added by the consideration of highly regulated expression pattern of S3.7 proteins, as evidenced from their high EV (see Text S1). The pattern of signal transduction between different parts of the network, therefore, may be regulated by modulating the expression levels of central proteins, thereby fine-tuning network behavior according to the conditions at hand. Therefore, it is tempting to speculate that the presence of rich clubs among highly dynamic proteins in the protein interaction networks of eukaryotes may be an evolutionarily selected mechanism of highly efficient yet regulated signal propagation across the network.

Since the initial observation of differential positioning of proteins in the network according to their expression profile based on a single metric (avPCC) [15], there has been some debate regarding whether the original observations by Han *et al* (2004) reflected an artifact of the specific network they used for their study [6], [7]. By utilizing a comprehensive survey of expression characteristics of proteins as well as of their immediate network localities in several datasets, our study confirms the notion of dynamic modularity in the eukaryotic protein interaction network. We show that a multidimensional analysis can resolve the discrepancy between these studies by offering a higher resolution view of the dynamic network organization. For example, the initial proposition of so-called “date” hubs to be central proteins by Han *et al* is refined in this study by showing that date hubs also contain highly modular static proteins as well as non-central organizer proteins. Moreover, most of the characteristics attributed to date hubs (like higher evolutionary rate, higher synthetic lethality rate, higher density of genetic interactions) turn out to be the characteristics of proteins in static modules, which, importantly, logically dissociates the notion of centrality from the variability in protein networks suggested earlier [18]. In addition, suggestion that the protein network lacks an organized pattern [6], [7] (and hence displays a disorganized highly inter-connected “stratus” pattern) is shown to be incorrect in this study by using a more comprehensive approach, even using the same dataset as in the original study of Batada *et al* [7]. We believe that further development of novel methodology for the analysis of biological networks is crucial for systems biology to be successful in discovering the complex fabric of life.

## Methods

### Datasets

Protein interaction network was compiled from studies of Krogan *et al* (2006) [13] (high quality binary interaction data) and Bader *et al* (2004) [12] (high quality interactions with a confidence cut-off of 0.65). For microarray compendium, we used the same dataset as in ref. 9.

### Deriving novel network metrics

First, we consider an |N|×|N| adjacency matrix *A* of the network with a node set *N* and an |N|×|N| expression correlation matrix *C*, constructed by calculating all pair-wise Pearson Correlation coefficients of expression profiles of genes using our microarray compendium. *A* is such that *A_i,j_* is 1 if and only if proteins *i* and *j* interact, and 0 otherwise. *C* is such that *C_i,j_* is the variance of the expression profile of gene *i* if *i* = *j*, otherwise it is the Pearson correlation coefficient of expression profiles of genes *i* and *j*. Variances of expression profiles of genes in the diagonal of *C* are normalized so that their values reflect their quantile in the whole distribution of variances [9] (i.e. these values range from 0 to 1).

#### Expression variance (EV)

As defined previously [9], expression variance (EV) of a protein is the statistical variance of its expression levels across all the conceivable conditions and reflects the extent of transcriptional regulation of a gene; so that a low EV indicates that the gene has a static expression pattern and therefore is not transcriptionally regulated, while a high EV indicates a highly regulated expression pattern.
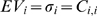



#### Neighborhood EV (nEV)

nEV is the average EV in the immediate neighborhood of a protein and is defined as 
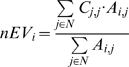



Neighborhood EV reflects the expression variances of a protein's neighbors in the network. We have shown that nEV can be particularly informative about a protein's location in the network [9]; low nEV of proteins being a strong indicator that the protein is located within densely connected modules in the network (i.e. set of proteins dedicated to a specific cellular process).

#### Variance in neighborhood EV (v^EV^)

This is variance of EV values in the neighborhood and is defined as:
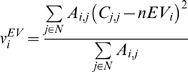
where *nEV_i_* is the neighborhood EV of gene *i*. This metric shows how variable the neighborhood of a protein is in terms of their EV, so that a neighborhood with high *v^EV^* would suggest that the neighborhood of the protein is composed of proteins with variable levels of regulation and may indicate that the protein is not located within a module.

#### Neighborhood Pearson correlation coefficient (nPCC)

This is the average expression correlation between neighbors of a protein and is defined as:
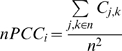
where *n* is the set of neighbors of protein *i*. nPCC is a dynamic equivalent of the clustering coefficient in social networks, and as opposed to *connectivity* coherence in social networks, it shows the extent of *expression* coherence in a protein's neighborhood. High nPCC indicates that the neighbors of the protein are highly co-regulated and that the protein is probably located within a dynamically regulated module (dynamic module) whose protein constituents are highly co-expressed.

#### 2nd neighborhood Pearson Correlation Coefficient (nPCC2)

This is the average nPCC among neighbors of a protein. nPCC2 reflects the extent of co-regulation in the second neighborhood of a protein. A protein with high nPCC2 but low nPCC is most likely to be located “just outside” of a dynamic module and interacting with one or more proteins inside the module.

#### Variance in the neighborhood Pearson Correlation Coefficient (v^PCC^)

This is variance in correlation between neighbors of a protein and is defined as:
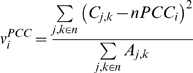



PCC variance (*v^PCC^*), reflects the variation in the co-regulation of proteins in the neighborhood. Like *v^EV^*, *v^PCC^* shows how variable the neighborhood is, but unlike *v^EV^*, *v^PCC^* also reports how similar or dissimilar the expression profiles of the neighbors are.

*Dynamic degree (yK): yK* is a dynamic equivalent of node degree in social networks. It is defined as the sum of its absolute PCC values with all proteins in the network, 
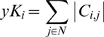
and reflects the number of proteins that it is co-regulated with. Formally, *yK* measures the size of the co-expression neighborhood of a protein, so that a protein with a high *yK* is probably a member of a gene expression program with many genes and therefore its expression may be tightly regulated, while a low *yK* would indicate that the protein's expression is not coupled to the expressions of other proteins in the network.

#### Neighborhood dynamic degree (*nyK*)

*nyK* is simply the average *yK* in a protein's neighborhood.

### *In silico* loss of function method for network connectivity analysis

*D* is an |N|×|N| matrix of shortest path distances between all node pairs in the network, where N is the set of nodes. Let *D* ***^x^*** be the distance matrix of a network formed by the deletion of a set 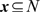 of nodes from the original network. A difference matrix 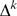 is such that 
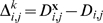
 if and only if *D_i.j_*≤*k*, and 0 otherwise. Value *k* denotes the distance of interest. For example if *k* = 2 (our case), differences in distances between node pairs that are 1 node apart in the original network are considered, so that if 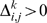, we conclude that some node(s) in ***x*** are directly linking nodes *i* and *j* in the original network. If all distances are to be considered, *k* = ∞ should be chosen.

We consider a null model for matrix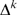, by performing 20 random deletions of |***x***| number of proteins with node degrees similar to ***x***. 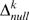 is such that 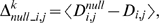
where 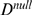 is the distance matrix of network formed by a random deletion of |***x***| number of nodes, of which there are 20. Normalized form of the difference matrix therefore becomes 
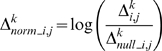
where each *i*, *j* position gives the amount of impact on the path length between nodes *i* and *j* relative to what would be expected by chance.

### Rich club coefficients

Rich club coefficient (*φ*) is defined as the density of interactions between nodes having node degrees larger than a specific value [17], 
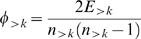



where *E_>k_* is the number of edges between, and *n_>k_* is the number of, nodes that have node degrees higher than *k*. We define rich club coefficient within the group as 
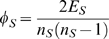
where *E_S_* is the number of edges between, and *n_S_* is the number of, nodes in group *S*. A null model is considered by randomly shuffling the positions of nodes at one side of the adjacency matrix 100 times (equivalent to random rewiring of each node's connections), and calculating the corresponding rich club coefficients at each time. Normalization of the within-group rich club coefficients against null model is performed by 
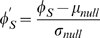
where *μ_null_* is the mean and *σ_null_* is the standard deviation of the distribution of the null model.

## Supporting Information

Text S1 Network plots of the dynamical classes. Plots were generated using gplot() function in sna package for R (http://erzuli.ss.uci.edu/R.stuff).
(0.25 MB PDF)

Figure S1 Detailed analysis of S3 subgroups.
(1.89 MB TIF)
