# Supplementary material for: Fine-Scale Dissection of Functional Protein Network Organization by Statistical Network Analysis
Source: PLoS One. 2009 Jun 24;4(6):e6017. doi: 10.1371/journal.pone.0006017 (PMC2699632; doi:10.1371/journal.pone.0006017)
Supplement: Text S1 — Network plots of the dynamical classes. Plots were generated using gplot() function in sna package for R (http://erzuli.ss.uci.edu/R.stuff). (0.25 MB PDF) [file pone.0006017.s001.pdf]

SUPPLEMENTARY INFORMATION for Komurov *et al*.

Clustering of S3 into 7 subgroups

Detailed dynamic profiles of each subgroup are seen in Figure S1.

S3.1: This subgroup has most of its values low just like S2, with the exception that it has relatively high v^{EV} and v^{PCC} (shown as varEV and varPC in the figure). Low nEV of this group indicates that proteins in this group are surrounded mostly by static (low EV) proteins, however their high v^{EV} and v^{PCC} suggest that their neighbors in the network have variable EV values and dissimilar expression profiles. This dynamic profile suggests that these proteins may be located at the boundaries of static modules. A network plot of a representative S3.1 protein (IES1, a subunit of the INO80 chromatin remodeling complex) shows that this protein links the module constituents to other proteins in the network (Fig. S2-A).

S3.2: This subgroup has relatively higher nEV values, but low v^{EV}, suggesting that these proteins are mostly interacting with high-EV (dynamic) proteins. However their relatively higher v^{PCC} also shows that neighbors of these proteins have dissimilar expression profiles, therefore indicating that S3.2 proteins are not found within modules. It follows that S3.2 proteins are coordinating functions of various dynamic proteins. An example to this subgroup is RHO1, a small GTPase involved in cytoskeleton signaling. This protein is mainly surrounded by dynamic proteins of various functions (Fig. S2-B).

S3.3: Dynamic profile of this subgroup is very similar to that of S3.1, with a subtle difference that this subgroup has higher *yK* and lower v^{PCC} values (Fig. S1). Higher *yK* values indicate that, unlike S3.1, proteins in this group may be co-expressed with other proteins in the network and thus may be a part of cellular gene expression programs. An example to this subgroup of proteins is KIN2, a protein kinase involved in the regulation of exocytosis (Fig. S2-C).

S3.4: The most striking feature of proteins in this subgroup is their high nPCC2 but low nPCC (Fig. S1), indicating that neighbors of these proteins have high nPCC values but low PCC with each other. This profile suggests that S3.4 proteins are found outside dynamic modules, perhaps bridging them. An example to this subgroup is SBA1, a co-chaperone that binds and regulates Hsp90 family chaperones. Its network plot shows that this protein is interacting with UFD1/NPL4/CDC48 complex involved in protein transport from ER to the cytosol, and also with a nuclear pre-ribosomal complex containing NUG1, NOG1, RIX1, etc… (Fig. S2-D).

S3.5: The most obvious feature of this subgroup is its highest v^{PCC} values. This indicates that neighbors of these proteins have highly dissimilar expression profiles, with probably negative co-expressions. Therefore, these proteins interact with some of their neighbors under one condition, and with the others under another condition, but never with both sets at the same time. A representative of this subgroup is BCY1, a regulatory subunit of cAMP-dependent protein kinase (PKA) (Fig. S2-E). It associates with either of the three catalytic subunits of PKA: TPK1, TPK2 or TPK3. TPK3 is negatively co-expressed with the other two subunits, indicating that TPK3 may have an opposing

function to the other kinases (Fig. S2-E). Indeed, TPK3 has been shown to inhibit pseudohyphal growth in yeast whereas TPK2 promotes it[1].

S3.6: Proteins in this subgroup have high EVs, high v^{EV} and high *yK* and avPCC (Fig. S1). Therefore, these proteins are dynamic, are co-regulated with other proteins in the network, they may also be co-regulated with their neighbors. However unlike dynamic modules, these proteins have high v^{EV} values, indicating that these proteins interact with proteins of various expression patterns. Proteins in this subgroup may be located at the boundaries of small dynamic modules. An example to this subgroup is MMS2, a protein involved in post-replication DNA repair (Fig. S2-F). The co-expression profile of its neighbors suggests that MMS2 is co-expressed with UBC13, RCN2 and SIP5. MMS2 is known to form a complex with UBC13, however the functional significance of its interactions with the RCN2 and SIP5 are not yet known.

S3.7: Proteins in this subgroup have high EV and v^{EV} values and moderate nEV values; the rest of its values are relatively low. This is the only subgroup where EV and *yK* values do not correlate (see Fig. S1). EV highly correlates with *yK* (Spearman's ρ = 0.68, $P < 1x10^{-16}$), which is not surprising as highly regulated proteins are more likely to be co-regulated with other proteins in the network. This high correlation is not a consequence of high variance *per se*, as Pearson correlation coefficient is defined as the ratio of covariance of two variables to their individual variances. The fact that S3.7 proteins are highly regulated but still are not co-regulated with many proteins in the network suggests that the expression profiles of these proteins are highly specific, which may be the case for master regulators of cellular processes in the cell. Accordingly, S3.7 contains proteins like FUS3 and SWI5, master regulators of pheromone response and cell cycle, respectively (Fig. S2-G).

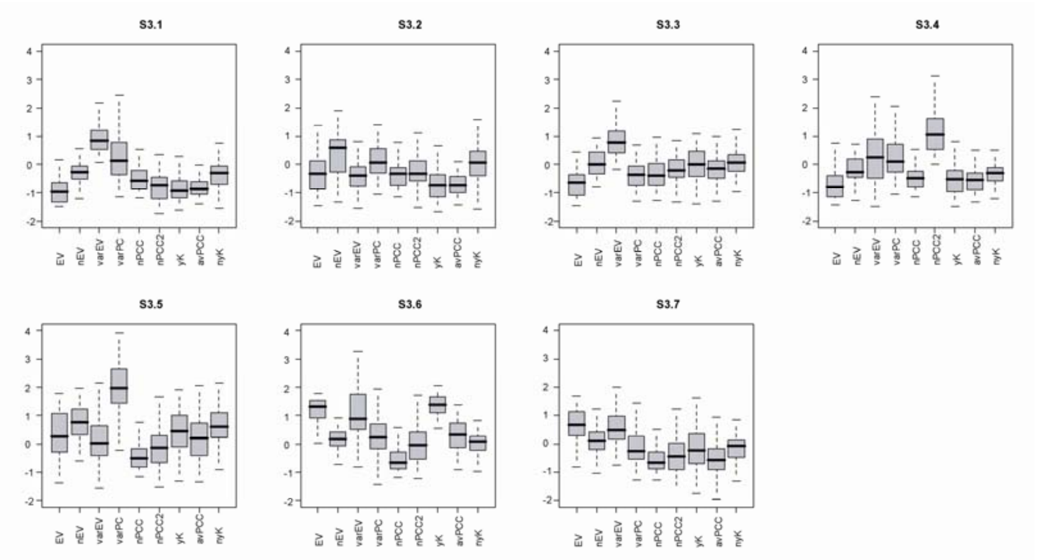

Figure S1. Dynamic profiles of each S3 subgroup. Y axes represent normalized values of each metric (see Methods).

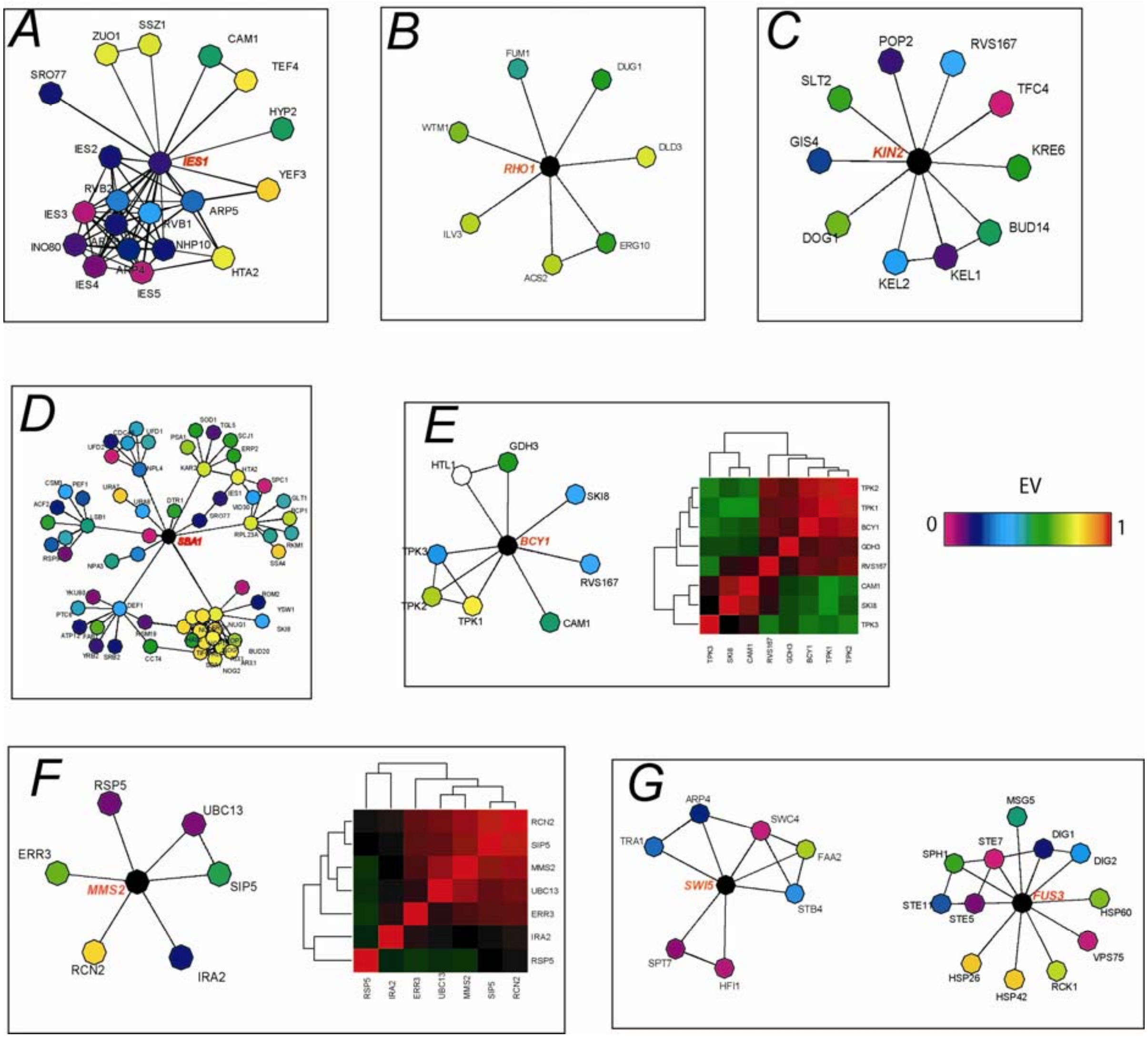

Figure S2. Representatives of each dynamic class. **A-G** show representative protein neighborhoods from subgroups S3.1 through S3.7, respectively. Heatmaps in **E** and **F** show expression correlation matrices of the neighbors of the respective proteins, where shades of green indicate strength of negative correlation while shades of red indicate strength of positive correlation. Nodes in the network plots are colored according to their EV (color key shown); nodes of interest (center nodes) are colored in black for convenience.

Reference

1. Robertson, L.S. & Fink, G.R. The three yeast A kinases have specific signaling functions in pseudohyphal growth. *Proc Natl Acad Sci U S A* **95**, 13783-7 (1998).
