# Supplementary figures and images for: Fine-Scale Dissection of Functional Protein Network Organization by Statistical Network Analysis
Source: PLoS One. 2009 Jun 24;4(6):e6017. doi: 10.1371/journal.pone.0006017 (PMC2699632; doi:10.1371/journal.pone.0006017)

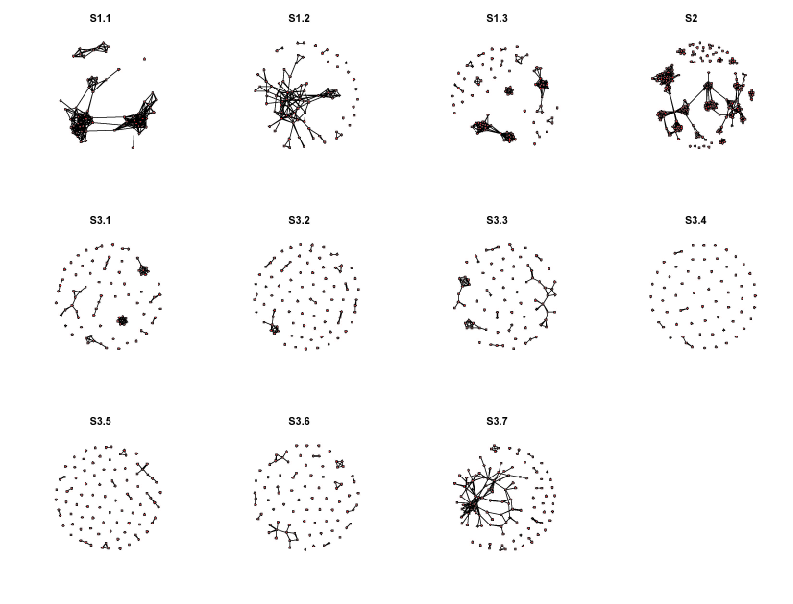

Supplement: Figure S1 — Detailed analysis of S3 subgroups. (1.89 MB TIF) [file pone.0006017.s002.tif]
